# Quantification of Ligand–Membrane Interactions Using DNP-NMR Relaxometry

**DOI:** 10.1021/acs.analchem.5c04414

**Published:** 2026-02-24

**Authors:** Chang Qi, Nirmalya Pradhan, Christian Hilty

**Affiliations:** Chemistry Department, Texas A&M University, College Station, Texas 77843, United States

## Abstract

The transverse relaxation rates (*R*_2_) for ^19^F spins from a small molecule ligand are measured in the presence of phospholipid vesicles with varied compositions and concentrations. The sensitivity of detection is enhanced by hyperpolarization using dissolution dynamic nuclear polarization (D-DNP), enabling measurement of *R*_2_ relaxation rates in single scans. The binding interaction is described as an equilibrium with a defined number of binding sites on the membrane. From the increase of *R*_2_ as a function of lipid concentration, a parameter (*f R*_2,b_)/*K*_D_ is calculated, which depends on the fractional number of binding sites per lipid (*f*), the relaxation rate (*R*_2,b_) of bound ligand, and the dissociation constant (*K*_D_) for each binding site. The relaxation rate of a bound ligand is modeled based on molecular motions, including rigid body tumbling, diffusion in the bilayer, wobble motions, and CF_3_ rotation. By estimation of *R*_2,b_ from chemical shift anisotropy and dipole–dipole relaxation, the dissociation constant *K*_D_/*f* is evaluated to greater than 10 mM for the ligand binding to 200 nm vesicles. The resulting values for the binding affinity were not significantly affected by the presence of cholesterol in the bilayer, but were reduced by vesicle aggregation. These data further demonstrate that *R*_2_ relaxation measurements using DNP hyperpolarization provide a means to detect ligand–membrane binding and kinetic parameters. In addition to *R*_2_, a set of other NMR derived parameters that are sensitive to molecular dynamics, such as *R*_1ρ_, diffusion and cross-relaxation, and spectroscopic techniques, such as Laplace NMR, are compatible with the hyperpolarized method. The measurement of membrane–ligand binding can facilitate applications for drug discovery and biomedical studies.

## Graphical abstract

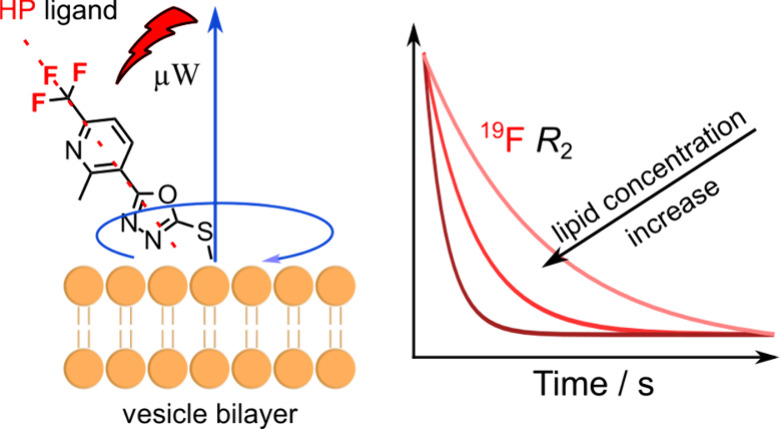

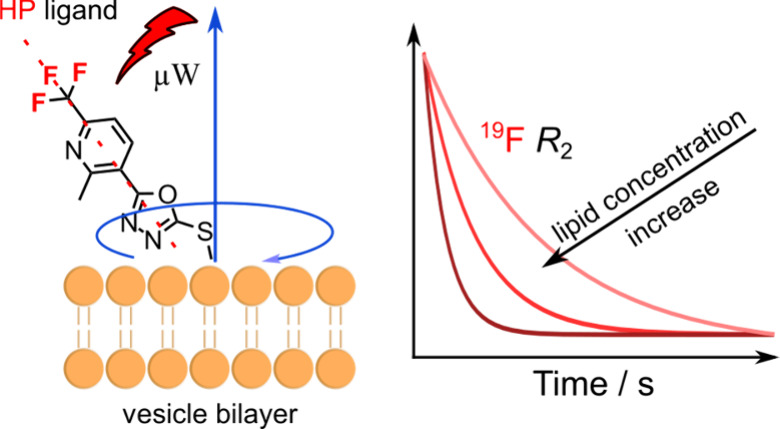

## Introduction

Cell membranes are increasingly recognized for their molecular diversity, as an environment where functional and pharmacological interactions occur not only with membrane proteins but also with lipid molecules and other components. The insertion of a small molecule drug modulates the function of the lipid membrane by affecting its physical properties such as thickness, fluidity, and charge, thus regulating the passive transport of materials across the lipid membrane.^1^ The membrane bilayers also play a role in the binding of small molecule inhibitors to membrane receptors, whereby molecules can initially bind to the membrane and subsequently diffuse toward the binding sites on the proteins.^2^ The interactions between ligand molecules and hormones, neurotransmitters, or ion channels that are distributed in the cell membrane but occupy only a minute fraction of its surface area are enhanced by the reduction in dimensionality to the two-dimensional membrane. Membrane binding also increases the formation of complexes bound to ligands with low water solubility. Recent studies reveal that ligand–membrane interactions are relevant to intercellular receptor–ligand binding as well. On this basis, understanding ligand–membrane binding provides essential guidance for designing new drugs.^3^

In characterizing ligand–membrane interactions, NMR provides the possibility to preserve the conformation and stereostructure of the ligand–membrane bound complex under physiological conditions. Solid-state NMR spectroscopy of ^1^H, ^31^P, and other nuclei has been applied to characterize the conformation of the bound ligand and the kinetics of the binding process.^4,5^ The interactions between a small molecule and the lipid membrane have also been investigated through the observation of NMR parameters including chemical shift difference,^6^ nuclear Overhauser effect,^7,8^ paramagnetic relaxation rates,^9^ as well as residual dipolar coupling in solution.^7^

Dynamic nuclear polarization (DNP),^10^ a technique that enhances the NMR signal by several thousandfold, can be used to decisively improve the detection limit in ligand binding studies. Hyperpolarization has been applied to facilitate the determination of kinetic parameters including binding affinity^11,12^ and characterization of ligand binding epitopes.^13–15^ Hyperpolarization-assisted ligand-observed NMR allows the screening of a ligand binding to immobilized proteins.^16^ The application of hyperpolarized ^19^F NMR enables background-free detection of a small molecule at a concentration of ∼μM in a single scan.

Here, we observe the relaxation rates of the hyperpolarized ^19^F spin on a small molecule ligand, which interacts with model membranes in small vesicles of different lipid compositions, to investigate the kinetic process of ligand–membrane binding. We then predict the contributions of the bound forms based on relaxation theory and use the results to estimate affinity ranges for the ligand binding to the membranes.

## Experimental Methods

### Vesicle Preparation

100% 1-Palmitoyl-2-oleoyl-*sn*-glycero-3-phosphocholine (POPC) (Avanti Polar Lipids, Alabaster, AL) vesicles as well as 70% POPC and 30% cholesterol (Avanti Polar Lipids, Alabaster, AL) vesicles were prepared for the experiments. An amount of 20 μmol of POPC lipids or 14 μmol of POPC lipids with 6 μmol of cholesterol were dissolved in 2 mL of CHCl_3_ and dried under N_2_ gas as a thin film. The lipids were further dried under vacuum overnight. After rehydrating with 5 mL of phosphate buffered saline (PBS; 137 mM sodium chloride, 2.7 mM potassium chloride, 10 mM sodium phosphate dibasic, and 1.8 mM potassium phosphate monobasic, pH 7.4) buffer, the mixture was thawed in a 55 °C water bath and frozen by liquid N_2_ 10 times. Extrusion was carried out using an extruder kit (Avanti Polar Lipids, Alabaster, AL) with 0.2 or 1 μm membranes (Whatman, Maidstone, UK) 11 times. A nonextruded sample was used directly after the freeze–thaw steps.

### DNP Experiments

A sample of 40 mM 2-methyl-3-(5-methylsulfanyl-[1,3,4]­oxadiazol-2-yl)-6-trifluoromethyl-pyridine (ICT5040; Aobious, MA) containing 15 mM 4-hydroxy-2,2,6,6-tetramethylpiperidine-1-oxyl (TEMPOL) radicals (Sigma-Aldrich, St. Louis, MO) was prepared in a mixture of DMSO-*d*_6_ and D_2_O at 4:1 v/v (Cambridge Isotope Laboratories, Tewksbury, MA). The sample (10 μL for gas-driven experiment and 2 μL for liquid-driven experiments with flow cell) was loaded into a HyperSense DNP polarizer containing a 3.35 T magnet (Oxford Instruments, Abingdon, UK) and irradiated with 100 mW microwaves at a frequency of 94.005 GHz and temperature of 1.4 K for 40 min. The hyperpolarized sample was dissolved with heated PBS buffer at 10 bar and rapidly transferred to the NMR spectrometer. Gas-driven injection was used to determine the DNP signal enhancement, applying forward and back pressures of 262 and 150 psi, respectively. A stabilization period of 500 ms was applied prior the NMR experiment.^17,18^ A 3D printed flow cell of 240 μL volume via liquid-driven injection was used in all of the other experiments. The flow cell was preloaded in an NMR instrument with a broad-band (BBO) probe (Bruker Biospin, Billerica, MA). In the liquid-driven injection, the dissolved DNP sample and vesicle samples were automatically loaded into sample loops. The volume of the nonhyperpolarized sample was determined by the 0.4 mL sample loop volume. Samples were subsequently injected into the flow cell by two high-pressure syringe pumps (Models 500D and 1000D, Teledyne ISCO, Lincoln, NE).^17^ The flow rates for the pumps were set to 80 and 200 mL/min, respectively. The corresponding injection times were 900 and 750 ms, applied with a simultaneous end point. The injection was followed by a 500 ms delay to stabilize the stopped fluid before NMR acquisition.

### NMR Spectroscopy

^19^F signals were collected on the 400 MHz NMR spectrometer at 298 K. A single-scan Carr–Purcell–Meiboom–Gill (CPMG) pulse sequence *p*1 – [τ – *p*2 – τ] × *n* (Figure S1) with an echo time 2τ of 1721.9 μs and loop number *n* of 2875 was used to measure the *R*_2_ relaxation rates.^11^ The *p*1 and *p*2 are hard pulses of π/2 and π flip angle applied with a pulse strength of *γB*_1_ = 47.6 kHz. The raw data of each experiment was reshaped into a two-dimensional data set according to its loop number and echo time using Python (Python Software Foundation, https://www.python.org). A sine-shaped window function was applied to every echo, and zero filling was performed to both ends of each echo with the number of zeroes added equal to the size of each echo. Every set of data points from one echo was Fourier transformed to obtain a spectrum containing the single peak for the ^19^F spin. The integrals of the peaks from all echoes were fitted to a single exponential decay versus the acquisition time to determine the *R*_2_ relaxation rate. The signal enhancement achieved by DNP was calculated by comparing the integrals of the ^19^F peaks obtained in the experiments with and without hyperpolarization.

## Results and Discussion

2-Methyl-3-(5-methylsulfanyl-[1,3,4]­oxadiazol-2-yl)-6-trifluoromethyl-pyridine (ICT5040) is a small molecule inhibitor of the G-protein coupled CXCR4 cell membrane receptor (Figure 1a).^19^ The interaction of this prototype drug molecule with 1-palmitoyl-2-oleoyl-*sn*-glycero-3-phosphocholine (POPC) vesicles was characterized. Figure 1b shows the hyperpolarized ^19^F NMR signal of ICT5040. The ^19^F signal is distinct, even in samples that comprise mixtures of biological molecules, where this nucleus is not abundant. The DNP signal enhancement for ^19^F in this molecule was estimated by injecting an aliquot of the hyperpolarized sample into an NMR tube. A signal enhancement of 768 ± 21 fold was determined by comparison with the spectrum of the sample after decay of the spin hyperpolarization.^20^

**Figure 1:**
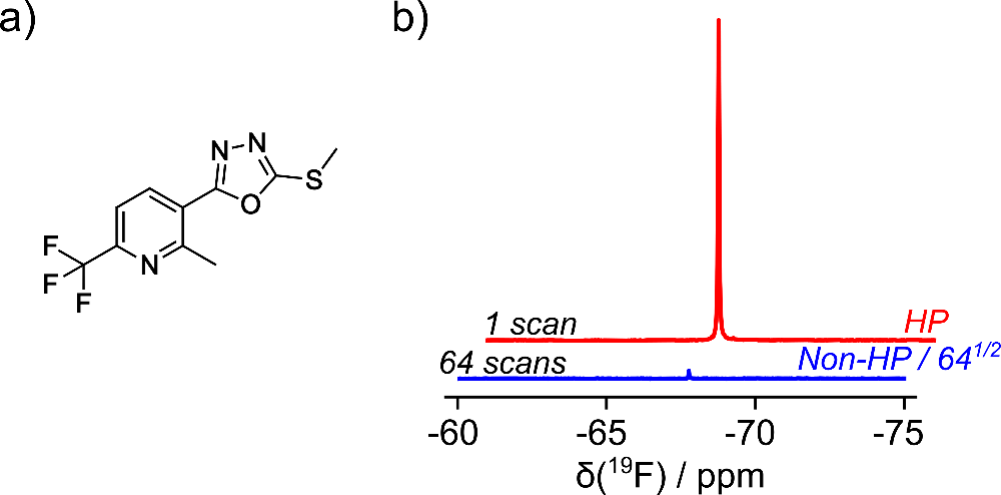
(a) Structure of ICT5040. (b) ^19^F spectrum of 0.32 mM DNP hyperpolarized ICT5040 (HP; red) compared to the same sample measured in 64 scans after decay of hyperpolarization (Non-HP; blue). The vertical scale of the nonhyperpolarized spectrum is reduced by a factor of 64^1/2^ = 8 to match noise levels.

Different relaxation rates were observed for the molecules in the presence of lipid vesicles of varying sizes and compositions. In Figure 2a, the presence of POPC vesicles extruded through a 200 nm membrane resulted in a faster *R*_2_ decay than for the free molecule, indicating that the molecule interacts with the lipid. The increase in *R*_2_ for the ^19^F spins was quantified by varying the lipid concentration for each of the vesicle types (Figure 2b). A linear dependence of the observed relaxation rate *R*_2,obs_ on the lipid concentration was seen in all cases, with a vertical axis intercept in agreement with *R*_2,*f*_. This behavior is explained by a simple model for the binding of the ligand molecule to available binding sites on the vesicles.

**Figure 2:**
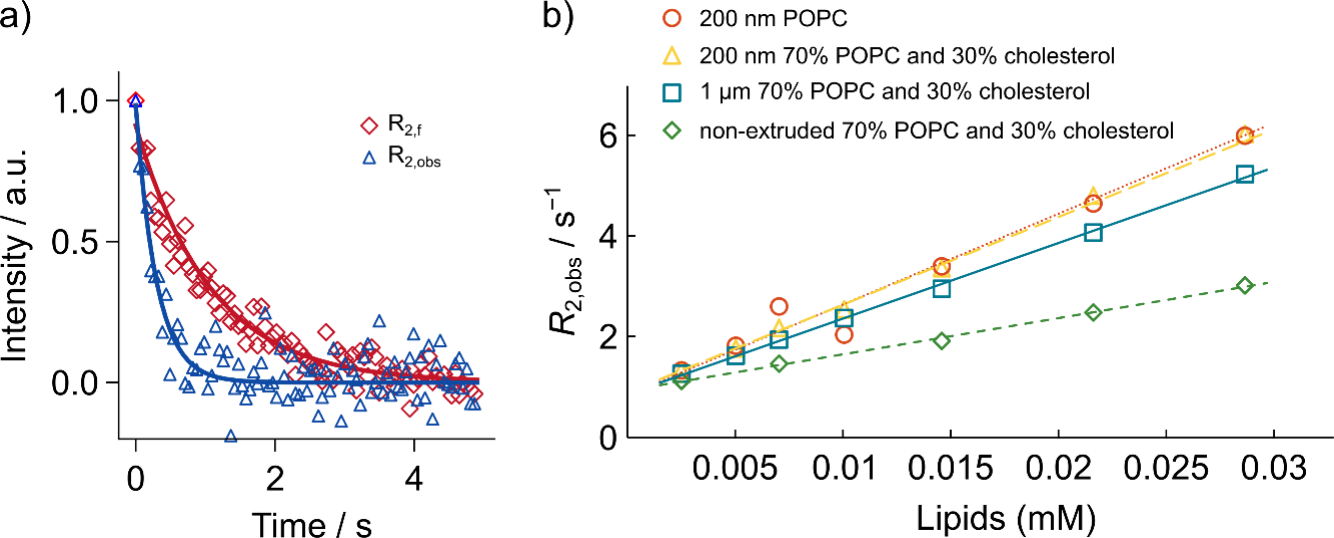
(a) Data points and fitted curves for *R*_2_ relaxation measurements of the ^19^F spin obtained in the absence (open diamond, *R*_2,f_ = 0.93 s^–1^) and presence (open triangle, *R*_2,obs_ = 3.37 s^–1^) of 0.015 mM 200 nm POPC vesicles where [L]_t_ = 33 μM. The data was acquired with a CPMG pulse sequence with an echo time of 1.7 ms in a single scan. In the figure, only every 32nd data point is plotted for the sake of clarity. (b) *R*_2_ relaxation rates of hyperpolarized ^19^F on ICT5040 measured with the vesicles indicated. The fitting equations of each line are *R*_2,obs_ = 179.84­[Lipids] + 0.84 (dotted line), *R*_2,obs_ = 173.75­[Lipids] + 0.90 (long dashed line), *R*_2,obs_ = 149.77­[Lipids] + 0.86 (solid line), and *R*_2,obs_ = 71.786­[Lipids] + 0.93 (short dashed line) with *R* ^2^ = 0.998, 0.964, 0.999, and 0.997, respectively. The lipid concentration was calculated according to the dilution factor in SI.

The model describing the interaction assumes that the ligand molecule can bind to a number of binding sites on the vesicle (eqs S1–S4). This number is proportional to the lipid concentration *B*_t_ = *f V*_t_. Here, *B*_t_ is the concentration of the assumed ligand binding sites and *V*_t_ the concentration of vesicle forming lipids ([Lipids] in Figure 2b). The factor *f* indicates the number of binding sites per lipid molecule (whereby *f* can be larger or smaller than one). The fraction of the bound ligand is calculated from the chemical equilibrium between free and bound ligand molecules

1 $$X_{b}=\frac{K_{D}+fV_{t}+L_{t}-\sqrt{{\left(K_{D}+fV_{t}+L_{t}\right)}^{2}-4fV_{t}L_{t}}}{2L_{t}}$$

In this equation, *K*_D_ is the dissociation constant for a ligand molecule with a binding site and *L*_t_ is the total ligand concentration. This equation corresponds to a well-known equation for binding found by solving the equations describing the equilibrium concentrations of free and bound species.^21^ Here, the factor *f* is added, which arises from the multisite model described above.

In the limit of fast exchange, the spin–spin relaxation rate is the average of the free and bound rates

2 $$R_{2,\mathrm{obs}}=X_{b}R_{2,b}+\left(1-X_{b}\right)R_{2,f}$$

It is noted that fast exchange is an assumption that is not trivially fulfilled; however, it appears reasonable for the presumed nonspecific binding between the bilayer and the small molecule ligand.

The dependence of *X*_b_, and consequently of *R*_2,obs_, on *V*_t_ according to eqs 1 and 2 exhibits saturation behavior (Figure S2). Based on the observed linear dependence of *R*_2,obs_ on *V*_t_, it can be concluded that the lipid concentration is sufficiently low for an initial slope approximation to be valid. The slope of *R*_2,obs_ at *V*_t_ = 0 under these conditions is

3 $$\frac{dR_{2,\mathrm{obs}}}{dV_{t}}=\frac{f\left(R_{2,b}-R_{2,f}\right)}{K_{D}+L_{t}}$$

with *R*_2,b_ and *R*_2,f_ denoting the spin–spin relaxation rates of bound and free ligands, respectively. When *L*_t_ ≪ *K*_D_ and *R*_2,f_ ≪ *R*_2,b_, the slope becomes (*f R*_2,b_)/*K*_D_.

Equation 3 indicates that *R*_2,b_ and *f*/*K*_D_ cannot be independently determined from the slope in Figure 2b. In the following, we compare this parameter under the assumption that *R*_2,b_ is independent of vesicle type and subsequently discuss an estimate for *R*_2,b_ based on prevalent relaxation mechanisms.

First, the interaction of ICT5040 in vesicles with or without cholesterol was investigated. Adding cholesterol to vesicle membranes is known to increase their viscosity and affect the transition temperature (*T*_m_). The *T*_m_ for POPC is −2 °C.^22^ The presence of 30% cholesterol may slightly affect the *T*_m_ and will make the phase transition less abrupt,^23^ but the measurements for vesicles with or without 30% cholesterol all take place in the liquid phase of the bilayer at room temperature. The addition of 30% (v/v) cholesterol also did not significantly affect the size or volume of vesicles as determined by dynamic light scattering (DLS) analysis (Figures S3 and S4, Tables S3 and S4).

Comparing the observed *R*_2_ of ICT5040 at the same lipid concentration, we found that the relaxation rates were not significantly different for 200 nm POPC vesicles with or without 30% cholesterol. For example, the *R*_2,obs_ values were determined as 1.77 ± 0.03 s^–1^ and 1.83 ± 0.05 s^–1^ using 5 μM 100% POPC and 70% POPC with 30% cholesterol, respectively. Here, the errors were calculated from the individual fits of the relaxation rates.

The (*f R*_2,b_)/*K*_D_ values fitted from the experiments with POPC vesicles that contain or do not contain 30% cholesterol were 174 s^–1^·mM^–1^ and 180 s^–1^·mM^–1^, respectively (Figure 3). Generally, the addition of cholesterol hinders diffusion of the lipids in bilayers. However, it does not appear to significantly affect the motions of the ligand molecule that cause the relaxation.

**Figure 3:**
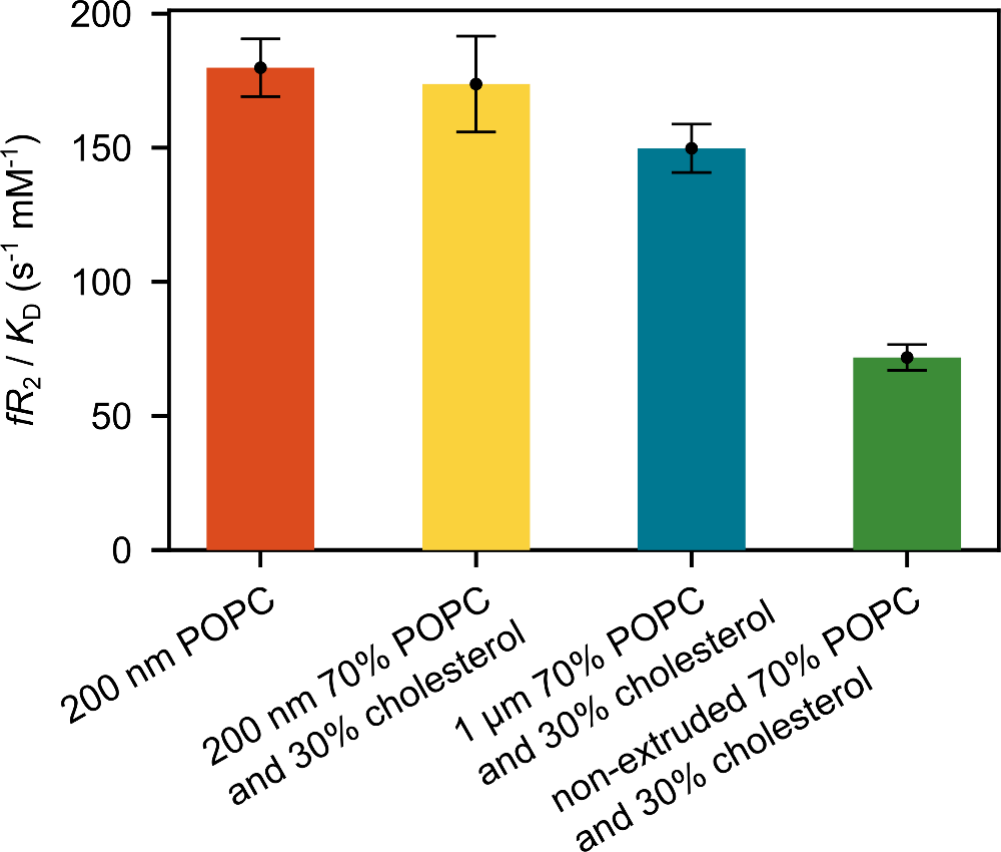
Quantity (*f R*_2,b_)/*K*_D_ determined from the initial slope of the titrations of hyperpolarized ICT5040 with POPC vesicles. The vesicles were extruded as described in the legend. The error bars were calculated from a 5.6% error in lipid concentration (Figure S3) and fitting errors of the *R*_2,obs_ curves.

Second, we characterized the dependence of observed relaxation rates on the size of vesicles. Figure 3 shows that POPC vesicles with 30% cholesterol extruded with a 200 nm membrane, a 1 μm membrane, and without extrusion exhibit progressively smaller (*f R*_2,b_)/*K*_D_. Regardless of the extrusion process, the largest number of vesicles has a size of 100 nm (Figure S3 and Table S1). However, extrusion with membranes of larger pore size results in some larger vesicles that occupy more volume, as seen in Figure S4c. The observed trend in (*f R*_2,b_)/*K*_D_ is contrary to the increase in *R*_2,obs_ that would be expected if the molecule were rigidly bound to structures of increasing size. Based on the observed change in (*f R*_2,b_)/*K*_D_, the vesicles extruded through the larger pores therefore exhibit weaker apparent binding or a smaller number of accessible binding sites evidenced by a smaller factor *f*. This behavior may be explained by larger vesicles containing a smaller accessible surface area per unit weight, which could, in part, be due to multilamellar features or aggregation behavior.

The initial slope of *R*_2,obs_ measured with increasing lipid concentration, (*f R*_2,b_)/*K*_D_, is the ratio of *R*_2,b_ and the binding parameter *K*_D_/*f*. Because the titration does not reach a high lipid concentration, where *R*_2,obs_ reaches a plateau as shown in Figure S2, in the linear regime at low concentration, the binding parameters and relaxation rates of the bound ligand cannot be determined independently. In the following, we discuss estimates for *R*_2,b_ based on relaxation theory. Once the *R*_2,b_ range is known, the range of binding affinities of the molecule for the membrane can be predicted accordingly.

A vesicle as a rigid sphere exhibits a correlation time τ_c_ of several hundred microseconds according to the Stokes–Einstein equation (eq S13). The lateral diffusion over the curved vesicle surface introduces a dependence on a transverse diffusion coefficient.^24,25^ The transverse diffusion for the small molecule is expected to be similar or faster than that of the lipid molecule, which was described as 1.9 × 10^–11^ m^2^ s^–1^ for POPC.^26^ For a vesicle of 200 nm diameter, this diffusion coefficient results in a reduced effective correlation time of τ_v_ = 43 μs (eq S15).^27,28^ The dipolar interaction between the ^19^F and the closest averaged ^1^H at a distance of 3.01 Å and intrafluoromethyl relaxation with a distance of 2.25 Å between fluorine spins results in *R*_2,b(DD)_ = 1.4 × 10^5^ s^–1^. The calculated chemical shift anisotropy for the molecule is Δσ = −56 ppm,^29^ which would contribute *R*_2,b(CSA)_ = 6.7 × 10^4^ s^–1^.

Additional motions of the ligands reduce the relaxation rates. In the following, the motion of the entire ligand in the membrane and the fast rotation of the CF_3_ group are considered. The corresponding correlation times and order parameters are included in a model-free treatment of the relaxation.^30,31^ The motion of the entire molecule may be described as a wobble motion with a typical diffusion coefficient *D*_w_ = 1/(6τ_w_) ≈ 10^7^–10^9^ s^–1^.^32^ The order parameter for a DPPC lipid molecule in a 100 nm vesicle was reported as ∼0.6 in the liquid phase.^28^ The bilayer of the POPC vesicles is also in the liquid phase at the experimental temperature of 298 K.^22^ For fluorescent probes in DPPC or POPC lipid membranes, the order parameter varied from 0.8 to 0.4 among the gel and liquid phases.^33^ The order parameter for the small molecule should be lower than that for a lipid. On the other hand, a model-free treatment of CF_3_ linked to a protein side-chain resulted in an order parameter and correlation time as low as 0.1 and 10^–9^ s, respectively.^34^ On the basis of these values, we consider *S*_*w*_^2^ of 0.1–0.4 and τ_w_ of 10^–9^–10^–8^ s for ICT5040 in the membrane.

For the CF_3_ rotation, the order parameter was estimated by using a computer simulation. The unlabeled carbon atom does not carry a nuclear spin, and the dominant interaction is with an adjacent hydrogen atom. The autocorrelation function was calculated using a time and ensemble average^35^ of random traces generated from jumps between three sites,^36^ resulting in *S*_*r*_^2^ = 0.54 for an F–H pair and *S*_*r*_^2^ = 0.24 for F–F (Figure S5). The latter matches a theoretically expected value of 1/4.^37^ In analogy to CH_3_, a correlation time τ_r_ = 10^–11^ s is assumed for the CF_3_ group.^38^ With these parameters, dipole–dipole relaxation results in an *R*_2,b(DD)_ of 3.6 × 10^3^ s^–1^ to 1.5 × 10^4^ s^–1^ (eqs S17–S19). The *R*_2,b(CSA)_ is 6.7 × 10^3^ s^–1^ to 2.7 × 10^4^ s^–1^, whereby the symmetrized chemical shift tensor implicitly includes fast CF_3_ rotation.

With eq 3 and *L*_t_ ≪ *K*_D_, the resulting *K*_D_/*f* value is between 57 mM and 230 mM. These values are assuming that the exchange between free and bound ligands is fast. An exchange contribution to *R*_2,obs_ due to slower exchange would lead to an underestimation of the *K*_D_ value. For other vesicles that were extruded through a 1 μm membrane or nonextruded, 92% or 67% of the vesicles are of the size 100 nm, while larger vesicles are present (Table S2). Nevertheless, the experimental (*f R*_2,b_)/*K*_D_ value became smaller. This may be due to the aggregation of large vesicles, as discussed above, which would reduce the number of accessible binding sites, *f*.

The change in the *R*_2_ relaxation of ICT5040 in different vesicles can be compared with line widths of lipid molecules. Figure S6 shows the ^1^H NMR spectra for different types of vesicles used in the experiments. The ^1^H line width was obtained for peaks from three regions, −N­(CH_3_)_3_ from the headgroup, −CH_2_ from the long chain, and −CH_3_. Some narrow signals that are not likely part of the bilayer are not considered in the analysis. The ^1^H line width measured for the−CH_2_ peak in the 100% POPC sample is about 78 Hz (Table S5). The presence of 30% cholesterol further broadens the ^1^H line width. This behavior is consistent with a reduction in the mobility of lipid molecules in the vesicle bilayer due to interactions with cholesterol. By contrast, the observed relaxation rate for the ligand that binds to vesicles with or without cholesterol does not show a significant change, suggesting that the mobility of the ligand in the vesicle membrane is only slightly affected by interactions with cholesterol.

The above differences illustrate the necessity of directly measuring ligand relaxation rates. The *R*_2_ parameter is interesting for the measurement of weak interactions at low concentrations because it is strongly influenced by binding, and the observed signal is averaged between free and bound forms. Even a small fraction of the total ligand in the bound form can produce a detectable change in the signal. The observation of the total hyperpolarized ligand signal distinguishes this method from other biophysical techniques that are based on measurement of the less abundant bound ligand complex, which may render the detection of weak interactions with dissociation constants in the mM range challenging.

The DNP-assisted relaxometry method allows the detection of ligand–membrane interactions for low concentrations of the ligand. A signal enhancement of 1000-fold results in a submicromolar detection limit, which mimics a physiological concentration. The single scan experiment is measured with a signal-to-noise ratio that would require approximately 2 weeks of averaging in conventional NMR. DNP simplifies the observation of a small amount of ligand by making hyperpolarized signals equivalent to or larger than the background signals. Although the ^19^F signal measured here is not affected by background signals, the polarization enhancement can be important for resolving signals from abundant spins such as ^1^H or ^31^P. In conventional NMR, signal overlap can be severe in the presence of large amounts of membrane lipid.

NMR relaxation is among the most sensitive observables that depend on molecular dynamics. The *R*_2_ parameter is strongly influenced by microsecond to millisecond exchange events. The existence of an extensive body of relaxation theory developed since the advent of NMR spectroscopy facilitates the interpretation of relaxation data. Spin hyperpolarization is becoming more widely available for biological molecules.^39^ New applications of relaxation theory extend the utility of hyperpolarization for dynamic measurements in biomolecular NMR spectroscopy.

The same measurement of *R*_2_ relaxation may be further applied to study the binding of ligands to membrane proteins in vesicles or possibly in cells. Since the structure of membrane proteins is more rigid compared to phospholipids in the membrane bilayer, the *R*_2,b_ for the ligands that bind to membrane proteins should be much larger compared to that for a ligand–membrane bound complex, but the number of binding sites would be lower.

Other NMR observables beyond *R*_2_ are compatible with the described method. In the presence of slow exchange dynamics, *R*_1ρ_, *R*_2_ dispersion, or self-diffusion, the latter of which can be measured isotropically or anisotropically, can show an association with lipid structures or molecular complexes. Cross-relaxation is also highly sensitive to binding to rigid complexes, which is exploited in transfer nuclear Overhauser effect (tr-NOE) experiments.^16,40^ The method is compatible with Laplace-NMR (LNMR), which analyzes the dynamic parameters by the time domain decay of signals and resolves multiple decay rates. Applications of ultrafast LNMR, where two-dimensional spectroscopy is capable of correlating these parameters, are emerging.^41,42^ A combination of these measurements may provide dynamic information on interactions in heterogeneous systems comprising membranes and membrane constituents beyond the binding affinity.

## Conclusions

The ligand–membrane interaction was studied by observing the transverse relaxation rates of a small molecule ligand that binds to vesicle bilayers at varied lipid concentrations. The observed relaxation rates can be interpreted in the context of relaxation theory to obtain information on affinity and the number of binding sites in the ligand–vesicle binding equilibrium. The determination of model parameters assist in the determination of ligand–membrane protein interactions for drug discovery by quantifying the off-target contribution to *R*_2_ relaxation that is due to lipid binding. They may also help in understanding the mechanism of drug delivery and action, where the association of a drug molecule in the membrane bilayer lowers the barrier of its binding to membrane proteins and provides an environment that could mediate the protein–ligand interactions.

## Supplementary Material



## Notes

CPMG pulse sequence, quantification of sample concentration in flow cell, binding equilibrium, dynamic light scattering, relaxation equations, ^1^H NMR signals of lipids (PDF)
