# Supplementary material for: Quantification of Ligand–Membrane Interactions Using DNP-NMR Relaxometry
Source: Anal Chem. 2026 Feb 24;98(9):6541–6. doi: 10.1021/acs.analchem.5c04414 (PMC12980485; doi:10.1021/acs.analchem.5c04414)
Supplement: Supplementary file 1 [file ac5c04414_si_001.pdf]

Supporting Information

Quantification of Ligand-Membrane Interactions Using DNP-NMR Relaxometry

Chang Qi, Nirmalya Pradhan and Christian Hilty*

Chemistry Department, Texas A&M University, College Station, TX 77843, USA

*email: chilty@tamu.edu

Table of Contents

CPMG Pusle Sequence........ S2
Quantification of Sample Concentration in Flow Cell........ S2
Binding Equilibrium........ S3
Dynamic Light Scattering........ S4
Relaxation Equations........ S7
    Modeling of Fluoromethyl Rotation........ S8
    Spectral Density and R_2 relaxation with Local Motions........ S9
^{1}H NMR Signals of Lipids........ S10
References........ S11

CPMG Pusle Sequence

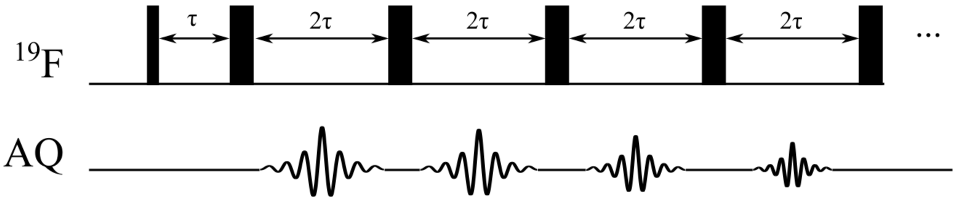

Figure S1. *Single-scan CPMG pulse sequence for measuring* R_2 *relaxation rates in a hyperpolarized experiment.*

The Carr-Purcell-Meiboom-Gill pulse sequence employed interleaved signal acquisition using a pulse program incorporating Bruker Biospin's pulse programming feature for signal acquisition with explicit generation of sampling intervals. The narrow and wide black bars represent 90 and 180 degree pulses, respectively (Figure S1). The echo time of 2τ comprises the signal acquisition time for each echo. Parameters are described in the main text.

Quantification of Sample Concentration in Flow Cell

During the injection, both hyperpolarized ligand and vesicle samples are diluted. The dilution factors for both samples were determined to calculate the final ligand and vesicle concentration. 1 mM DSS (sodium 2,2-dimethyl-2-silapentane-5-sulfonate-D_6) (Cambridge Isotope Laboratories, Tewksbury, MA) was loaded into the flow cell directly from the inlet tubing without injection to avoid dilution. A 2 uL sample of 200 mM DSS was loaded in the DNP sample cup instead of the ligand sample and injected into the flow cell without hyperpolarizing the sample. The signal integrals for the DSS sample that was loaded directly into the flow cell or from the injection were compared to obtain the dilution factor. The injection was repeated three times to calculate the average dilution factor. Similarly, the dilution factor for the vesicle sample was measured by three repeated injections where the vesicle sample was replaced by 0.4 mL 1 mM DSS.

Table S1. *Concentrations for the DSS in the flow cell from injections where 2 uL 200 mM DSS was used to replace the hyperpolarized sample. The concentration was calculated by comparing the peak integral with that from the 1 mM DSS loaded directly into the flow cell.*

trial	DSS concentration	Dilution factor	Average dilution factor
1	0.167 mM	1198	1188 ± 60
2	0.178 mM	1124	
3	0.161 mM	1242	

Table S2. *Concentrations for the DSS in the flow cell from injections where 0.4 mL 1 mM DSS was used to replace the vesicle sample. The concentration was calculated by comparing the peak integral with that from the 1 mM DSS loaded directly into the flow cell.*

trial	DSS concentration	Dilution factor	Average dilution factor
1	0.476 mM	2.101	1.98 ± 0.11
2	0.513 mM	1.949	
3	0.529 mM	1.890	

Binding Equilibrium

The binding mechanism for a ligand L that interacts with the binding sites B on vesicle is shown as below, where K_D is the dissociation constant for a ligand molecule with a binding site. K_D is assumed to be the same regardless how many binding sites occupied. LB is the bound complex of one ligand associated to one binding site.

$$L + B \underset{K_D}{\Leftrightarrow} LB \qquad \text{(S1)}$$

Mass balance equations were used for the calculation of the fraction of bound ligand.

$$[L]_t = [L] + [LB] \qquad \text{(S2)}$$

$$[B]_t = [B] + [LB] \quad , \textit{i.e.} \quad [V]_t = [V] + [LB]/f \qquad \text{(S3)}$$

where $[L]_t$ and $[V]_t$ are the total concentration of ligands and vesicle lipids, respectively. $[B]_t$ is the total concentration of binding sites. [L], [V], and [LB] are the concentrations of unbound ligand, unbound vesicle lipids, and bound complex. f represents the number of binding sites per lipid molecule (notice that f can be smaller or larger than 1).

The K_D is expressed by Eq. S4.

$$K_D = \frac{[L] \cdot (f \cdot [V])}{[LB]} \qquad \text{(S4)}$$

The fraction of bound ligand, X_b, in Eq. 1 of the main text is calculated by combining Eq. S1-S3. The Eq. 2 in the main text finds the observed relaxation rate from the weighted average of the free and bound relaxation rates, under the assumption of fast exchange between the free and bound forms. The resulting graph, shown in Figure S2, shows the familiar curvature due to saturation behavior. At low the concentration limit, the curve is approximated with a straight line with the slope in Eq. 3 in the main text.

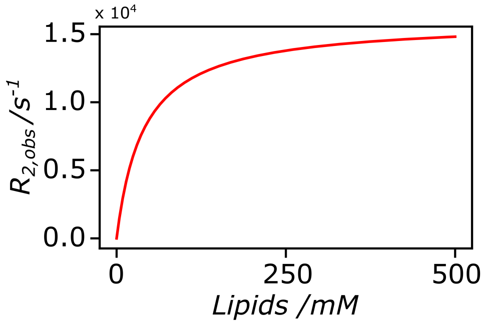

Figure S2. *$R_{2,obs}$ plotted against the lipid concentration $[V]_t$. Parameters are $[L]_t$ = 33 μM, K_D = 40 mM, f = 1. The R_2 relaxation rates for free and bound ligands are 0.92 s^{-1} and $1.6 \cdot 10^4$ s^{-1}, respectively.*

Dynamic Light Scattering

Dynamic light scattering (DLS) analysis was performed with a Zetasizer Nano (Malvern Panalytical Ltd, Malvern, UK) to measure the size distribution of vesicle samples. The acquired data were interpreted by a number-weighted distribution and volume-weighted distribution. The data analysis was performed by Zetasizer Nano software version 3.30 (Malvern Panalytical Ltd, Malvern, UK).

The number-weighted distribution represents the number of species with different sizes contained in the vesicle sample. When the sample contains N_a and N_b molecules with size *a* and *b*, respectively, the equation used to calculate the number-weighted distribution for the specie with size *a* is[1]

$$\%N_a = \frac{N_a}{N_a + N_b} \times 100\% \quad \text{(S5)}$$

In Figure S3, a single peak corresponding to a diameter of approximately 100 nm is visible. However, different extrusion processes produce different populations comprising a smaller number of vesicles of larger size. The volume-weighted distribution in Figure S4 calculates the relative volume occupied by vesicles with multiple sizes, which distinguish the larger vesicles from the majority that are smaller in size.

$$\%V_a = \frac{N_a a^3}{N_a a^3 + N_b b^3} \times 100\% \quad \text{(S6)}$$

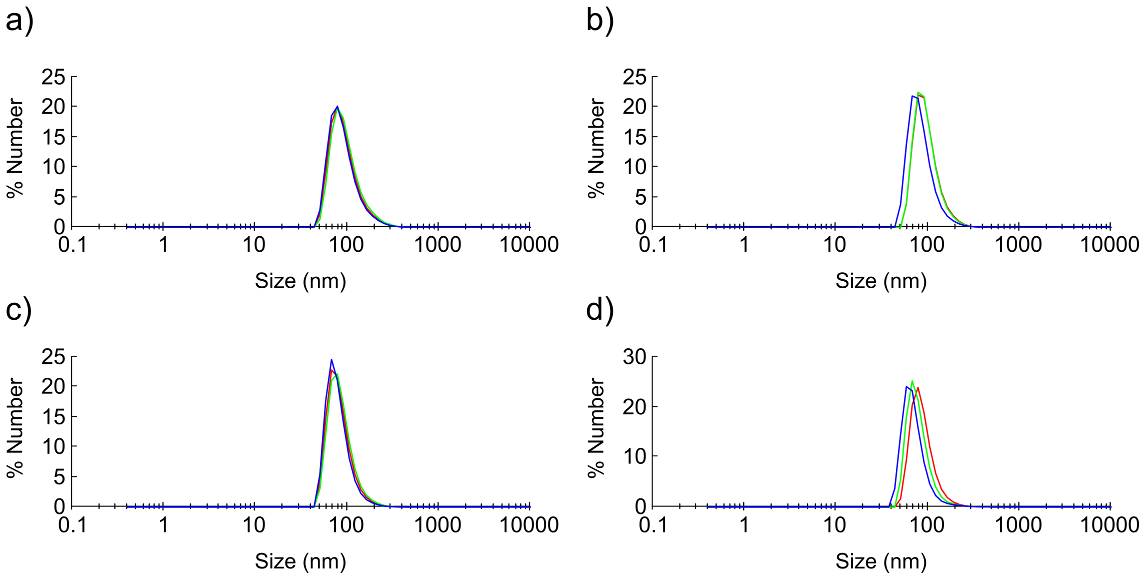

Figure S3. *The number-weighted distribution measured by DLS for 400 μM a) 100% POPC vesicles extruded through a 200 nm membrane, 70% POPC and 30% cholesterol vesicles extruded through b) a 200 nm membrane, c) a 1μm membrane and d) without extrusion in PBS buffer. Each figure shows three repeated measurements from the same sample.*

Table S3. *The corresponding number-weighted distribution with standard deviation averaged from three repetitions shown in Figure S3.*

Sample	Membrane	Size (nm)
100% POPC	200 nm	96 ± 40
70% POPC + 30% Cholesterol	200 nm	99 ± 34
70% POPC + 30% Cholesterol	1 μm	85 ± 30
70% POPC + 30% Cholesterol	NA	90 ± 35

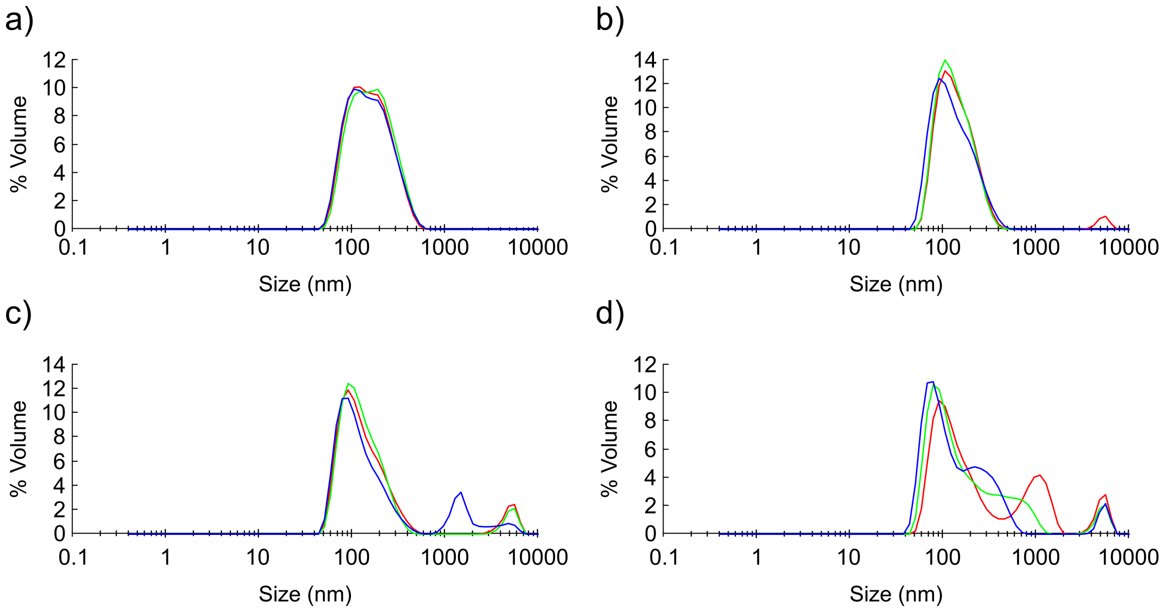

Figure S4. *The volume-weighted distribution measured by DLS for 400 µM a) 100% POPC vesicles extruded through a 200 nm membrane, 70% POPC and 30% cholesterol vesicles extruded through b) a 200 nm membrane, c) a 1µm membrane and d) without extrusion in PBS buffer. Each figure shows three repeated measurements from the same sample.*

Table S4. *The corresponding volume-weighted distribution with standard deviation averaged from three repetitions shown in Figure S4.*

Sample	Membrane	Size (nm)	% Volume	Size (nm)	% Volume	Size (nm)	% Volume
100% POPC	200 nm	170 ± 88	100	—	—	—	—
70% POPC + 30% Cholesterol	200 nm	146 ± 64	97.2	5230 ± 750	2.8	—	—
70% POPC + 30% Cholesterol	1 µm	137 ± 76	92.1	4950 ± 910	7.9	—	—
70% POPC + 30% Cholesterol	NA	142 ± 81	67.8	990 ± 310	24.3	5130 ± 810	7.8

Relaxation Equations

Two primary relaxation mechanisms, dipole-dipole (DD) interaction and anisotropic chemical shielding (CSA), were considered in the estimation of R_2 for the bound complex. Their contribution to the transverse relaxation rates are shown by Equations S7, S8 and S14.[2,3]

The dipole-dipole relaxation of ^{19}F spins considered a contribution from the nearest proton, as well as of the methyl group ^{19}F spins themselves.

$$R_{2,(\mathrm{DD})} = R_{2,\mathrm{HF(DD)}} + 2 \cdot R_{2,\mathrm{FF(DD)}} \tag{S7}$$

The total R_2 relaxation rate is the sum of the DD and CSA contributions,

$$R_2 = R_{2,(\mathrm{DD})} + R_{2,(\mathrm{CSA})} \tag{S8}$$

The individual dipole-dipole relaxation contributions were calculated as

$$R_{2,\mathrm{HF(DD)}} = \frac{1}{20} b_{\mathrm{HF}}^2 \{4J(0) + J(\omega_H - \omega_F) + 3J(\omega_F) + 6J(\omega_H) + 6J(\omega_H + \omega_F)\} \tag{S9}$$

$$R_{2,\mathrm{FF(DD)}} = \frac{3}{20} b_{\mathrm{FF}}^2 \{3J(0) + 5J(\omega_F) + 2J(2\omega_F)\} \tag{S10}$$

where the spectral density function for isotropic random motion is

$$J(\omega) = \frac{\tau_c}{1 + (\omega \tau_c)^2} \tag{S11}$$

and

$$b_{\mathrm{XY}} = \frac{\mu_0 \hbar \gamma_X \gamma_Y}{4 \pi r_{\mathrm{XY}}^3} \tag{S12}$$

whereby X and Y can be hydrogen or fluorine spins, as indicated in Equations S9 and S10.

For the calculation of intra-fluoromethyl relaxation, cross-correlation effects were not considered, as these would be beyond the scope of the model-free analysis. Therefore, the relaxation was calculated as twice the contribution from individual spin pairs.[4]

The $\mu_0 = 4\pi \cdot 10^{-7}$ Hm^{-1} is the vacuum permeability, $\hbar = 1.055 \cdot 10^{-34}$ Js is the reduced Plank constant, $\gamma_H = 2.68 \cdot 10^8$ s^{-1}T^{-1} and $\gamma_F = 2.52 \cdot 10^8$ s^{-1}T^{-1} are the gyromagnetic ratios of ^{1}H and ^{19}F, and $\omega = 2\pi \cdot \gamma$. The distance between the ^{19}F to ^{1}H spins and ^{19}F to ^{19}F spins are r_{HF} and r_{FF}, respectively. A geometry optimization was performed using the B3LYP DEF2-SVP basis set in ORCA 6.0.1-avx2 to find the conformation of ICT5040 molecule.[5] The r_{HF} = 3.01 Å was calculated as the average $\langle r_i^{-3} \rangle^{-1/3}$ between three ^{19}F nuclei and the closest ^{1}H. The second nearest ^{1}H is at a distance of more than 5 Å. Since Equations S9 and S10 depend on the sixth power of r, the corresponding dipolar effect is less than 5% compared to that from the closest proton.

The distances between fluorine spins in the fluoromethyl group (perpendicular to the methyl group rotation axis) are r_{FF} = 2.25 Å. This distance is included in Equation. S10.

For a rigid rotor, the rotational correlation time τ_c can be calculated from the Stokes-Einstein equation

$$\tau_c = \frac{4\pi\eta R^3}{3kT} \quad \text{(S13)}$$

where $\eta = 8.9\cdot10^{-4}$ Pa·s is the dynamic viscosity of the solvent, *i.e.* water.[6] R = 75 nm is the average of the vesicle radii from the DLS the measurements (Table S4), $k = 1.38\cdot10^{-23}$ J K^{-1} is Boltzman's constant and T = 298 K is the temperature. For these parameters, τ_c = 380 μs, and $R_{2(DD)} = 1.2\cdot10^6$ s^{-1}.

The relaxation due to chemical shift anisotropy is

$$R_{2,(CSA)} = \frac{1}{45}(\gamma_F B_0 \Delta\sigma)^2 [4J(0) + 3J(\omega_F)] \quad \text{(S14)}$$

In the calculation of $R_{2(CSA)}$, $\Delta\sigma$ is the chemical shift anisotropy.[7] The chemical shift anisotropy of each ^{19}F was calculated using their shielding tensor, obtained from computation in ORCA 6.0.1-avx2 with the DEF2-TZVPP basis set and averaged as reported for CF_3 rotation.[5,8] The averaged chemical shift anisotropy of ^{19}F is $\Delta\sigma$ = -56 ppm results in $R_{2(CSA)}$ on the order of $5.9\cdot10^5$ s^{-1}.

For a lipid molecule that laterally diffuses along the curved shell of the vesicle membrane, the transverse diffusion contributes to the effective correlation time

$$\tau_v = \left(\tau_c^{-1} + \tau_t^{-1}\right)^{-1} = \left(\frac{3kT}{4\pi\eta R^3} + \frac{6D}{R^2}\right)^{-1} \quad \text{(S15)}$$

D is the transverse diffusion coefficient, which for lipids from POPC vesicles is $1.9\cdot10^{-11}$ m^{2}s^{-1}.[9] The lateral diffusion time, τ_t was introduced with τ_c (Equation S15) and calculated effective transverse diffusion correlation time τ_v = 43 μs. Some difference is expected for the D parameter of a small molecule that laterally diffuses in vesicle bilayers, as discussed in the main text. Under this condition, the calculated $R_{2(DD)}$ and $R_{2(CSA)}$ are on the order of $5.8\cdot10^3$ s^{-1} and $6.7\cdot10^4$ s^{-1} respectively.

Modeling of Fluoromethyl Rotation

The order parameter for the rotating CF_3 group interacting with the nearest aromatic proton was determined using a computer simulation. In the simulation, random walk sequences were generated for the fluorine atoms jumping between three positions, each time by rotating through an angle of 2π/3 (Figure S5a). These jumps are in accordance with Tropp's N-site jump model for N=3.[10] The jump probability per simulation step in each direction was chosen arbitrarily as 0.1. This parameter together with the step size would determine the correlation time for the motion, but is irrelevant for the measurement of the order parameter from the correlation function. The number of time steps in each sequence was 10,000. The autocorrelation function was calculated as[11]

$$C(n_t)=\left\langle \frac{3\cos^2\theta(n_t+n_\tau)-1}{r^3(n_t+n_\tau)} \frac{3\cos^2\theta(n_t)-1}{r^3(n_t)} \right\rangle_{n_\tau} \tag{S16}$$

Here, θ is the angle that the time-varying distance vector between the hydrogen and the jumping fluorine atom form with the z-axis and r is the length of the distance vector. The time variables are represented in terms of the number of simulation steps n_t and n_τ for the time shift t in the autocorrelation and the time averaging parameter τ. The time averages were calculated up to a maximum shift of 100 simulation steps. To account for the random orientation of the molecules with respect to the magnetic field, 1,000 simulations were performed for randomly oriented molecules. The random orientations of the molecule were generated with a uniform distribution on the surface of a sphere. The final correlation function was obtained as the ensemble average of each simulation. The order parameter S_r^2 = 0.54 for the relaxation mechanism was read from the tail of the correlation function normalized with the value at zero time (Figure S5b).

The order parameter for the intra-fluoromethyl relaxation is different, as this mechanism depends on the F-F distances in the methyl group. These distances are perpendicular to the methyl group rotation axis. The order parameter was estimated by the same calculation described above, using the corresponding distance vectors (Figure S5c). The resulting value matches with an expected result of ¼ by comparison with ref.[4] .

The normalized function is approaching the order parameter as its asymptotic value. The calculations were performed using Python including the rdkit, py3Dmol, numpy, matplotlib and scipy packages.

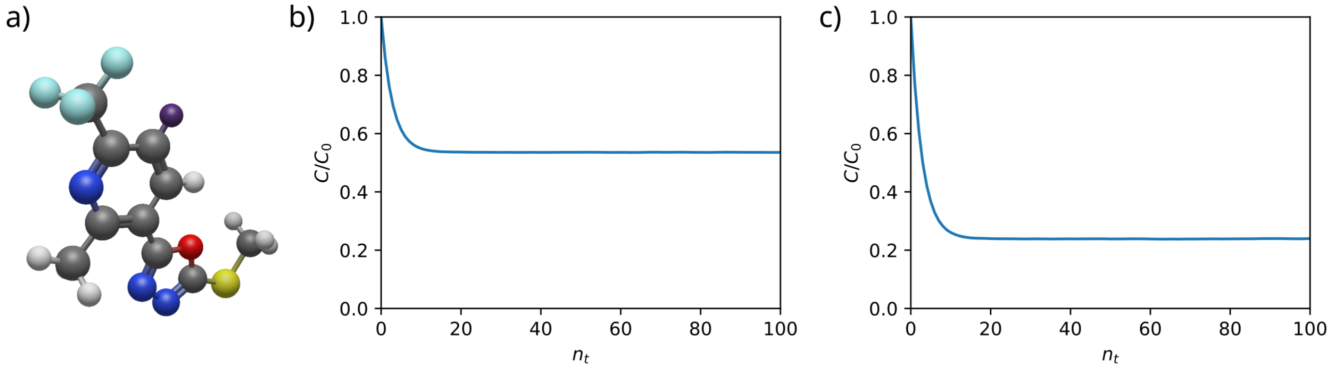

Figure S5. *a) Structure of the ICT5040 molecule used for the simulation. The three fluorine atoms are colored in cyan (top left), and the nearest aromatic proton is indicated in purple. b) Time and ensemble averaged autocorrelation function from the simulation of the rotating CF_3 group interacting with the nearest aromatic proton. The function is normalized to 1 at a time of 0. The n_t is the time difference in the autocorrelation in terms of the number of simulation steps in the random walk sequence. The order parameter read from the tail region of the function is S_r^2=0.54. c) Calculation as in (b), but for the intra-fluoromethyl relaxation resulting in S_r^2=0.24.*

Spectral Density and R_2 relaxation with Local Motions

Local motions of the molecule can be accounted for using the extended model free treatment. The spectral density is[12]

$$J_{ext}(\omega)=S_w^2 S_r^2 \frac{\tau_v}{1+(\omega\tau_v)^2}+S_w^2(1-S_r^2)\frac{\tau_r'}{1+(\omega\tau_r')^2}+(1-S_w^2)\frac{\tau_w'}{1+(\omega\tau_w')^2} \quad \text{(S17)}$$

with the correlation times

$$\tau_w'=\left(\tau_v^{-1}+\tau_w^{-1}\right)^{-1} \quad \text{(S18)}$$

and

$$\tau_r'=\left(\tau_v^{-1}+\tau_r^{-1}\right)^{-1} \quad \text{(S19)}$$

Here, S_w^2 and τ_w represent a motion of the entire small molecule in the membrane, which may be described as a "wobble-in-cone" motion. S_r^2 and τ_r describe the CF_3 rotation. When not considering a motion, the corresponding order parameter can be set to 1. If one order parameter equals 1, the equation reduces to the Lipari-Szabo equation.[13] If both are equal to 1, the rigid body spectral density results.

Considering the motion of the entire ligand with $S_w^2=0.1$, τ_w = 10^{-9} s and methyl group rotation with $S_r^2=0.54$ and τ_r = 10^{-11} s, $R_{2(DD)}$ = $3.1\cdot10^{2}$ s^{-1}. With $S_w^2=0.4$ and τ_w = 10^{-8} s, $R_{2(DD)}$ = $1.3\cdot10^{3}$ s^{-1}.

With the motion of the entire ligand with $S_w^2=0.1$, τ_w = 10^{-9} s, $R_{2,(CSA)}$ = $6.7\cdot10^{3}$ s^{-1}. With $S_w^2=0.4$ and τ_w = 10^{-8} s, $R_{2,(CSA)}$ = $2.7\cdot10^{4}$ s^{-1} s^{-1}. As $\Delta\sigma$ is averaged, the spectral density for calculating $R_{2(CSA)}$ is without rotational motion ($S_r^2=1$).

^{1}H NMR Signals of Lipids

^{1}H spectra were acquired for vesicle samples used in the experiments. Lorentzian fitting was performed to obtain the line widths for the three proton peaks from the vesicle spectra.

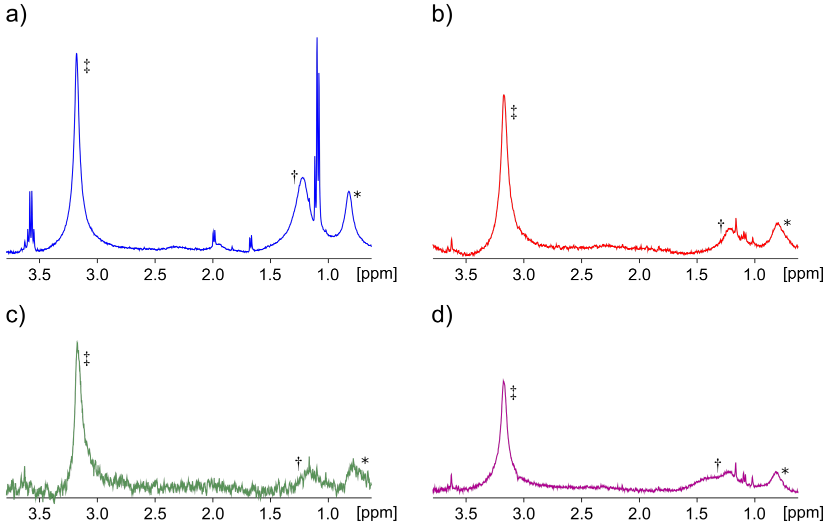

Figure S6. *^{1}H NMR spectra recorded without hyperpolarization, of 4 mM a) 100% POPC vesicles extruded through a 200 nm membrane, 70% POPC and 30% cholesterol vesicles extruded through b)*

*200 nm, c) 1µm membranes and d) without extrusion, in PBS buffer. -$N(CH_3)_3$, -CH_2 and -CH_3 peaks are labeled with ‡, † and *. b) and d) are scaled up by 2-fold, c) is scaled up by 8-fold.*

Table S5. *Fitted ^{1}H line widths for the three proton peaks shown in Figure S6.*

Sample	Membrane	-$N(CH_3)_3$ ^{1}H line width (Hz)	-CH_2 ^{1}H line width (Hz)	-CH_3 ^{1}H line width (Hz)
100% POPC	200 nm	22.84 ± 0.12	78.24 ± 0.16	42.04 ± 0.16
70% POPC + 30% Cholesterol	200 nm	29.92 ± 0.12	85.96 ± 0.76	66.12 ± 0.20
70% POPC + 30% Cholesterol	1 µm	24.68 ± 0.36	—	—
70% POPC + 30% Cholesterol	NA	30.04 ± 0.16	—	—

References

(1) Stetefeld, J.; McKenna, S. A.; Patel, T. R. Dynamic Light Scattering: A Practical Guide and Applications in Biomedical Sciences. *Biophys. Rev.* **2016**, *8* (4), 409–427. https://doi.org/10.1007/s12551-016-0218-6.

(2) Cavanagh, J.; Fairbrother, W. J.; Palmer, A. G.; Rance, M.; Skelton, N. J. Chapter 5 - Relaxation and Dynamic Processes. In *Protein NMR Spectroscopy (Second Edition)*; Cavanagh, J., Fairbrother, W. J., Palmer, A. G., Rance, M., Skelton, N. J., Eds.; Academic Press: Burlington, 2007; pp 333–404. https://doi.org/10.1016/B978-012164491-8/50007-5.

(3) Kowalewski, J.; Mäler, L. *Nuclear Spin Relaxation in Liquids: Theory, Experiments, and Applications*; Series in Chemical Physics; Taylor & Francis, 2006.

(4) Werbelow, L. G.; Marshall, A. G. Internal Rotation and Methyl Proton Magnetic Relaxation for Macromolecules. *J. Am. Chem. Soc.* **1973**, *95* (16), 5132–5134. https://doi.org/10.1021/ja00797a006.

(5) Weigend, F.; Ahlrichs, R. Balanced Basis Sets of Split Valence, Triple Zeta Valence and Quadruple Zeta Valence Quality for H to Rn: Design and Assessment of Accuracy. *Phys. Chem. Chem. Phys.* **2005**, *7* (18), 3297. https://doi.org/10.1039/b508541a.

(6) Wu, Y.; Štefl, M.; Olzyńska, A.; Hof, M.; Yahioglu, G.; Yip, P.; Casey, D. R.; Ces, O.; Humpolíčková, J.; Kuimova, M. K. Molecular Rheometry: Direct Determination of Viscosity in Lo and Ld Lipid Phases via Fluorescence Lifetime Imaging. *Phys. Chem. Chem. Phys.* **2013**, *15* (36), 14986–14993. https://doi.org/10.1039/C3CP51953H.

(7) Rüdisser, S. H.; Goldberg, N.; Ebert, M.-O.; Kovacs, H.; Gossert, A. D. Efficient Affinity Ranking of Fluorinated Ligands by ^{19}F NMR: CSAR and FastCSAR. *J. Biomol. NMR* **2020**, *74* (10–11), 579–594. https://doi.org/10.1007/s10858-020-00325-x.

(8) Neese, F. The ORCA Program System. *WIREs Comput. Mol. Sci.* **2012**, *2* (1), 73–78. https://doi.org/10.1002/wcms.81.

(9) Gaede, H. C.; Gawrisch, K. Lateral Diffusion Rates of Lipid, Water, and a Hydrophobic Drug in a Multilamellar Liposome. *Biophys. J.* **2003**, *85* (3), 1734–1740. https://doi.org/10.1016/s0006-3495(03)74603-7.
(10) Tropp, J. Dipolar Relaxation and Nuclear Overhauser Effects in Nonrigid Molecules: The Effect of Fluctuating Internuclear Distances. *J. Chem. Phys.* **1980**, *72* (11), 6035–6043. https://doi.org/10.1063/1.439059.
(11) Richarz, R.; Nagayama, K.; Wüthrich, K. Carbon-13 Nuclear Magnetic Resonance Relaxation Studies of Internal Mobility of the Polypeptide Chain in Basic Pancreatic Trypsin Inhibitor and a Selectively Reduced Analogue. *Biochemistry* **1980**, *19* (23), 5189–5196. https://doi.org/10.1021/bi00564a006.
(12) Clore, G. M.; Driscoll, P. C.; Wingfield, P. T.; Gronenborn, A. M. Analysis of the Backbone Dynamics of Interleukin-1.Beta. Using Two-Dimensional Inverse Detected Heteronuclear Nitrogen-15-Proton NMR Spectroscopy. *Biochemistry* **1990**, *29* (32), 7387–7401. https://doi.org/10.1021/bi00484a006.
(13) Lipari, G.; Szabo, A. Model-Free Approach to the Interpretation of Nuclear Magnetic Resonance Relaxation in Macromolecules. 1. Theory and Range of Validity. *J. Am. Chem. Soc.* **1982**, *104* (17), 4546–4559. https://doi.org/10.1021/ja00381a009.
